# Optimizing palliative care education nationwide: a practice example from The Netherlands

**DOI:** 10.1177/26323524241298288

**Published:** 2024-11-20

**Authors:** Ingrid van Zuilekom, Jojanneke Thiesen – van Staveren, Marijke Dericks-Issing, Marieke van den Brand, Harmieke van Os-Medendorp, Suzanne Metselaar

**Affiliations:** Research Group Smart Health, School of Health, Saxion University of Applied Science, Postbus 70.000, 7500 KB Enschede, The Netherlands; Amsterdam UMC, Location VUmc, Postbus 7057, 1007 MB Amsterdam, The Netherlands; Amsterdam UMC, Amsterdam, The Netherlands; Amsterdam UMC, Amsterdam, The Netherlands; Department of Anesthesiology, Pain and Palliative Medicine, Radboud UMC, Nijmegen, The Netherlands; Faculty of Health, Sports and Social work, Inholland University of Applied Sciences, Amsterdam, The Netherlands; Spaarne Gasthuis Academy, Hoofddorp, The Netherlands; Department of Ethics, Law and Humanities, Amsterdam UMC, Amsterdam, The Netherlands

**Keywords:** appreciative inquiry, co-creation, generalist palliative care, (initial) education, interprofessional collaboration, participatory approach

## Abstract

**Background:**

Every healthcare professional (HCP) in the Netherlands is expected to provide palliative care based on their initial education. This requires national consensus and clarity on the quality and goals of palliative care education and accessible education opportunities nationwide. These requirements were not met in the Netherlands, posing a major obstacle to improving the organization and delivery of palliative care. Therefore, a program, Optimizing Education and Training in Palliative Care (O^2^PZ), was established to improve palliative care education on a national level.

**Objectives:**

The main task of the O^2^PZ program from 2018 to 2021 was to implement and improve palliative care education in initial education for nursing and medical professionals. The program’s ultimate goal was that every HCP be sufficiently educated to provide high-quality generalist palliative care.

**Design:**

The O^2^PZ program consists of four projects to improve and consolidate generalist palliative care education nationwide.

**Methods:**

All projects used a participatory approach, that is, participatory development, implementation, and co-creation with stakeholders, mainly HCPs and education developers. Appreciative inquiry was used to assess, improve, and integrate existing local palliative care education initiatives.

**Results:**

(1) Establishment of an Education Framework for palliative care for all HCPs, including an interprofessional collaboration model; (2) optimization of palliative care education in the (initial) curricula of vocational education institutions and (applied) universities; (3) establishment of an online platform to disseminate materials to improve palliative care education; and (4) installment of seven regional palliative care education hubs, of which one hub was devoted to pediatric palliative care, as well as one national hub.

**Discussion:**

We discuss some lessons learned and challenges in accomplishing the goals of the O^2^PZ program in 2018–2021 and address how these challenges were dealt with. We maintain that co-creation with stakeholders at policy, organizational, and operational levels, as well as ongoing communication and collaboration, is essential to consolidating and implementing results.

**Conclusion:**

Over the past 4 years, we have improved generalist palliative care education nationwide for all HCPs through four projects in which we collaborated closely with stakeholders. This has resulted in more attention to and implementation of palliative care in education, a national Education Framework for palliative care, including an interprofessional collaboration model, an online platform for palliative care education, and palliative care education hubs covering all regions of the Netherlands.

## Plain language summary

Optimizing palliative care education in The Netherlands

The innovative aspect of this article is that we explored how a national program called “Optimizing Education and Training in Palliative Care” (O^2^PZ), which was funded by the Dutch government, was installed. The program was led by an interdisciplinary and interprofessional group of educators, HCPs, and researchers from several institutions in the Netherlands. The encompassing aim of the O^2^PZ program is that every HCP in the Netherlands has the right competencies to provide high-quality palliative care and that there will be more regional and national direction with regard to providing good palliative care education for all healthcare professionals. Our article will address several interconnected projects and results from the O^2^PZ program in 2018–2021. This includes (1) A comprehensive national Education Framework for Palliative Care (with a model of interprofessional collaboration), (2) Optimizing Palliative Care education, (3) An online education platform, and (4) A network of regional “education hubs” with nationwide coverage. Several lessons can be learned from these efforts to develop, implement, and consolidate palliative care education nationwide. We will also discuss some significant challenges and how they were dealt with effectively. This may be useful to others who seek to improve palliative care education in their country. Finally, we will address current follow-up studies to elaborate on and consolidate the results and impact of the 2018–2021 program.

## Background

Although significant efforts have been made over the last few years, palliative care provision in the Netherlands can still be improved significantly. Many people with palliative care needs still receive inappropriate or insufficient treatment and care.^1,2^ Studies report poor interdisciplinary teamwork, limited communication, and a lack of early identification of patients with palliative care needs and timely referral.^3–5^ Correspondingly, patients experience fragmentation and a lack of knowledge by healthcare professionals (HCPs) and coordination between HCPs and healthcare settings.^4,6,7^

The World Health Organization (WHO) has stated the importance of palliative care education for improving palliative care provision.^8^ For instance, according to the WHO, palliative care education should be embedded in all (nursing) programs. Yet, there is a lack of appropriate education and training in palliative care for HCPs in the Netherlands.^9–11^ Palliative care is sometimes hardly discussed or merely an elective course. This is problematic because, taking demographic developments into account,^12–14^ all (future) HCPs will increasingly have to provide palliative care.^15,16^ Furthermore, studies identified a lack of attention to complex symptom management, advance care planning (ACP), effective communication, and care coordination, which is also due to inadequate education and training in these subjects.^17–19^

These lacunas in education and training may exist because healthcare education for nursing and physician professionals predominantly focuses on a biomedically and medical task-oriented healthcare model and curative care rather than palliative care.^20,21^ This applies to all levels of graduate education (vocational, applied university, and university level) as well as to ongoing (postgraduate) education and training programs for HCPs who already work in practice. Yet, the tide is turning. Over the past decade, initiatives have been taken in many countries to include palliative care in nursing and medical curricula, for instance, through developing competency frameworks. The European Association for Palliative Care outlines which core competencies professionals should possess in a consensus-based white paper.^22^ In the Netherlands, a national palliative care program became part of the Dutch government’s health policy in 2007. The government aims to improve palliative care in such a way that all citizens in their final phase of life receive care and support that meets their—and their family’s—wishes and needs on a physical, psychological, social, and spiritual level.

A significant step forward toward more coherence and national consensus on palliative care quality standards was the 2017 publication of a National Quality Framework Palliative Care (NQFPC).^20,23^ The NQPFC defines the principles and criteria of high-quality palliative care’s various aspects or “domains.” It describes a mixed generalist-specialist palliative care model and articulates what this requires from all HCPs in the Netherlands. It is based on the international Gold Standards Framework.^24–28^ Various initiatives to improve palliative care education and training have been undertaken in line with this NQFPC. Most of these initiatives, however, pertain to efforts at a local and regional level and concern introductory and advanced courses in palliative care for nurses and physicians.^9,29^

### The O^2^PZ program

A 2016 knowledge synthesis on palliative care education concluded that, due to a lack of national direction, coherence, and collaboration, much of the efforts to improve the quality of palliative care education did not lead to actual and consistent changes in education practice.^30^ This led to the installation of a national program called Optimizing Education and Training in Palliative Care (O^2^PZ). The program consists of an interdisciplinary and interprofessional group of education professionals, HCPs, and policy-makers from several institutions in the Netherlands, and it is coordinated by Amsterdam University Medical Centers.^31^ ZonMw funds O^2^PZ. ZonMw programs and funds research and innovation in health, healthcare, and well-being, encourages using this knowledge, and highlights knowledge needs.^32^

The encompassing ambition of the O^2^PZ program is that every HCP has the right competencies to provide high-quality generalist palliative care, and that there is more national direction, collaboration, and consistency in palliative care education for all HCPs. Its main task is, therefore, to promote high-quality education and training throughout the country at all professional levels.^31^

**Figure 1.**
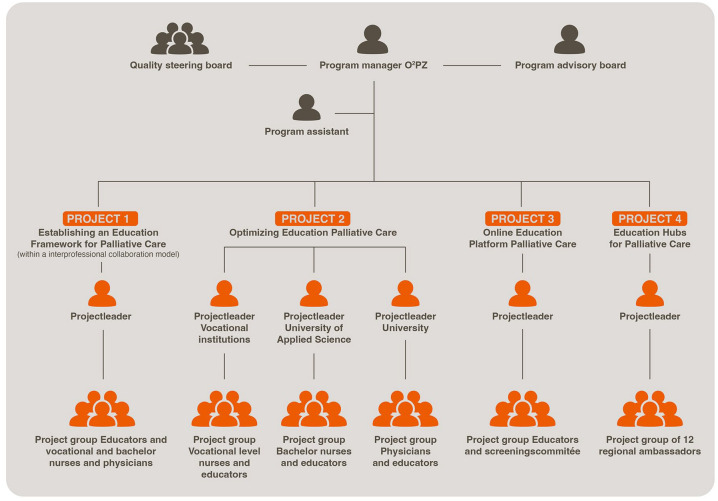
O^2^PZ organization chart 2018–2021.

Over 4 years (2018–2021), O^2^PZ conducted four projects (cf. Figure 1) to improve palliative care education and training nationwide in the Netherlands. This article reports on these four projects and their results. Furthermore, some major challenges and how they were dealt with (lessons learned) are discussed. Discussing this example from Dutch practice may be useful to others who seek to improve palliative care education and training in their country.

## Objectives

To accomplish the overarching aim of the O^2^PZ program, that is, improving palliative care education nationwide, the following tasks were set.

1. To develop an Educational Framework for palliative care education for nursing and medical professionals that describes the appropriate competencies to provide palliative care on all professional levels. To design a model to elucidate how the competencies described in the framework are complementary and what this means for interprofessional collaboration in palliative care.
2. To optimize existing education palliative care on all (initial) nursing and medical educational levels in line with both the education framework and the NQFPC, in order to ensure that (future) HCPs enter practice with the right competencies and can provide high-quality (generalist) palliative care.
3. To develop an online education platform devoted to palliative care education and training, on which, among other things, freely available educational materials can be found.
4. To create a network of regional education hubs across the Netherlands. The education hubs aim to be the “linking pin” between palliative care research, practice, and education, as well as between national innovation and standards and local and regional education and training initiatives.

## Design

A program manager, a quality steering board, a program advisory board, and, for each project, a project leader were established, from which further directions were given to the executing O^2^PZ program team. Four project teams were established in line with the four tasks described above. A communication expert was engaged to create broader awareness and support for the program and its objectives (social media, organizing conferences, news items on LinkedIn, and monthly newsletters). Figure 1 shows the organization chart of the O^2^PZ program during the 2018–2021 period.

## Method

Two methodologies were leading in the four O^2^PZ projects: a *participatory development approach* was mostly employed in projects 1 and 4, while projects 2 and 3 used *appreciative inquiry*. The fundamental idea of taking a participatory development approach is that if you want to create innovations with real-world impact—in this case, impact on the quality of education in palliative care—you should involve the envisioned end users as well as other relevant stakeholders in their development from the very start.^33^ Appreciative inquiry seeks to establish change and improvements by identifying and appreciating what works well and exploring how to improve rather than looking at problems and deficiencies. This method strengthens relationships and joint ownership in the process.^34,35^

### Establishing an Education Framework for (generalist) palliative care (project 1)

A project group was set up to develop a National Education Framework for palliative care that describes the competencies in terms of Entrustable Professional Activities (EPAs) needed to provide high-quality generalist palliative care at each level of education.^36^ Concretely, this development process entailed close collaboration and co-creation between education developers, HCPs, educators and trainers, students, and other stakeholders, such as education organizations on vocational,^37^ bachelor,^38^ and master levels.^39,40^ The O^2^PZ project leaders led this diverse group, who discussed and reflected on the competencies (knowledge, skills, attitudes, and behaviors) necessary for providing good palliative care on a generalist level.^9^ In doing so, they considered and compared the NQFPC^20,23^ with the 10 core competencies as described in a white paper of the European Association of Palliative Care.^22^ Validation efforts with HCPS and educators focused on ensuring that the complexity of the competencies aligned appropriately with each professional level. In addition to the Education Framework for individual HCPs, the project group jointly developed an interprofessional collaboration model to indicate the competencies of different professionals that are complementary in an interprofessional approach to palliative care.

### Optimizing palliative care education (project 2)

Appreciative inquiry was used to optimize assessed curricula with relevant stakeholders in 17 applied universities, 8 universities, and 35 vocational education institutions.^41^ These educational institutions currently determine the content of their curricula based on standards described by national education councils and associations of the nursing and medical professions. Yet, they have much freedom to choose a focus on specific subjects, and the way they deal with palliative care as a subject widely differs. An O^2^PZ project leader was appointed to facilitate improving existing palliative care education for all general HCPs. First, a contact person in each educational institution was appointed. Selection criteria for contact persons were having an affinity with and expertise in palliative care and the ability to influence the curriculum. Second, the project leader and contact persons made an inventory of the institution’s existing curriculum (subjects, course load, assignments, and assessments) to assess how palliative care is represented and where it is missing, using the Education Framework for palliative care of Project 1. From there, iterative steps were taken to improve the representation of palliative care. All contact persons from the participating educational institutions met online every 2 months to discuss and monitor the process. Through iterative rounds of co-creation, current education materials were (re)designed, tested, and evaluated.

### Developing an online education platform (project 3)

Appreciative inquiry was also used to establish an online education platform for palliative care. A comprehensive but (geographically) dispersed range of continuing (postgraduate) education in palliative care and many different educational materials were available in the Netherlands. There was no overview and quality assessment of what was offered, and, more often than not, descriptions of the learning objectives and required entry level were unclear. Thus, it was unclear where HCPs could go to improve their competencies and where teachers could find the best educational materials. For this purpose, O^2^PZ developed an online education platform for palliative care. We chose to do this in collaboration with the Foundation Palliative Care Netherlands (PZNL),^42^ which already had a platform called Palliaweb,^43^ an online platform for palliative care in general. A design of digital education platform was developed to allow users, such as education developers and teachers, to find and access a plethora of up-to-date materials, courses, and tools on palliative care education. Before being placed online, all educational materials were screened by an appointed screening committee consisting of palliative care (education) experts to assess and, if necessary, improve the materials’ quality.

### Creating a network of regional palliative care education hubs (project 4)

Palliative care education hubs were developed using a participatory approach. The leading question in this project was how to create more connections between regional educational institutions, the professional field, and research institutes. The Netherlands already has seven palliative care consortia,^43^ which are partnerships of regional care providers and organizations, expertise centers, and local palliative care networks. In these partnerships, the link with education was still lacking, which is why the idea arose in the advisory group to set up an education hub within these existing consortia. In a palliative care education hub, innovations from practice and research are introduced into education in palliative care so as to optimize this education. The education hub is meant to establish exchange, collaboration, and cohesion between educational institutions and other palliative care partners in the consortium. To achieve this, an ambassador was appointed to take a leading role in the education hub. The steering group selected these ambassadors based on their affinity with palliative care and their role as “linking pin” within the triad education, research, and practice.

## Results

Several products and results were established in the 2018–2021 period of the O^2^PZ program (see Table 1 for an overview).

**Table 1.** Results O^2^PZ program period 2018–2021.

Project	Results and output O^2^PZ 2018–2021	Method	Description
1. Establishment of an Education Framework for palliative care (including an interprofessional collaboration model)	Palliative care competencies for all nursing and medical professions were developed in co-creation. Also, the interprofessional collaboration competencies were developed.	Participatory development	The Education Framework for palliative care describes the competencies of palliative care generalists at all professional/educational levels. The interprofessional collaboration model describes six interprofessional activities in palliative care.
2. Optimizing palliative care education at the generalist level	Improved existing education on all nursing and medical professional levels for general palliative care.	Appreciative inquiry	To assess how palliative care is represented and where it is lacking in 17 applied universities, 8 universities, and 35 vocational education institutions.
3. Establishing an online education platform for palliative care	An accessible and findable education platform for all educators and HCPs.	Appreciative inquiry	An online education platform for palliative care was developed to disseminate materials to improve palliative care education. Including: A. Toolbox B. Elective courses in palliative care for vocational training C. Overview of continuing (postgraduate) education and training, D. Competency scan
4. Palliative care education hubs	Installment of a national and regional network.	Participatory development	Network of seven regional palliative care education hubs, a national hub entirely devoted to pediatric palliative care, one national hub, and one hub on pediatric palliative care, led by 12 ambassadors. The hubs implement innovations, developments, and activities in palliative care education in their region.

We describe the results for each project.

HCP, healthcare professional.

### Education Framework for palliative care (project 1)

A main outcome of the O^2^PZ program is an Educational Framework for palliative care that spans all levels of healthcare education.^44^ It delineates the knowledge, skills, attitudes, and behaviors as competencies of all HCPs caring for patients with (general) palliative care needs, including their relatives.^45,46^ In alignment with the international literature and the NQFPC, the Education Framework for palliative care describes the palliative care approach.^20,23^ The Education Framework for palliative care is intended for professionals frequently involved with palliative care patients in their care setting but for whom palliative care is *not* the main focus of their clinical practice.^8,22,47,48^

Additionally, this framework includes a practical tool for educators to assess, refine, and enhance their palliative care education and training. Although it can be utilized for both initial and continuing education and training, the central emphasis of the framework is on defining competencies and EPAs along with their corresponding behavioral expressions for graduate education. The relation between competencies and behavior was explicitly described to elucidate the concrete expectations of HCPs when providing palliative care in clinical practice.^44^

**Figure 2.**
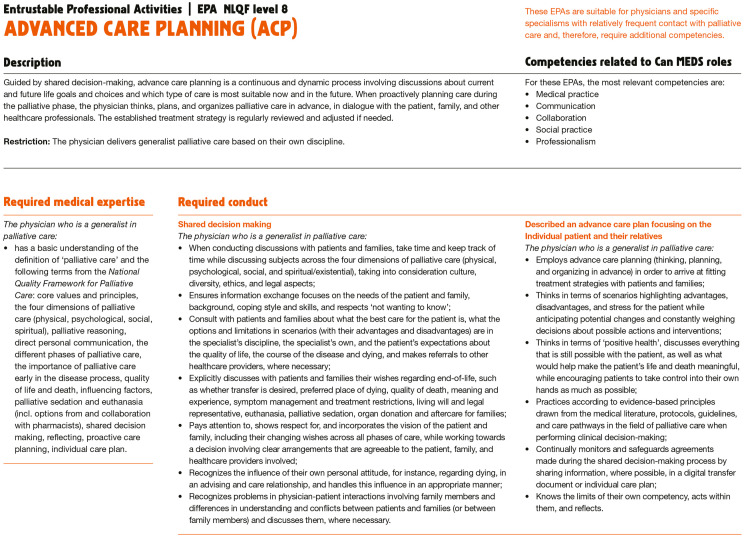
Illustration of an EPA for a physician. EPA, Entrustable Professional Activity.

Importantly, the components in the Educational Framework for palliative care are recommendations, not mandatory inclusions in the curriculum. The framework is (being) implemented in the curricula of (applied) universities and vocational education institutions and is also found on the digital education platform.^44^ Figure 2 shows an example of the Educational Framework: a physician’s EPA in ACP.^36^

### Interprofessional collaboration model

An important element of the Education Framework is the interprofessional collaboration model.

**Figure 3.**
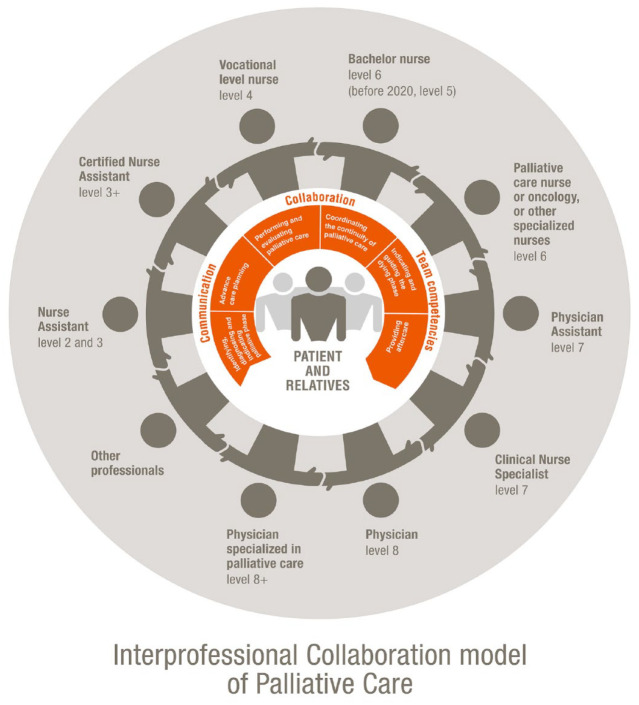
Interprofessional collaboration model palliative care.

Essential skills for interprofessional collaboration are crucial to providing good palliative care (Figure 3).^49,50^ Accordingly, a palliative advisory team consists of different disciplines. To provide some background, in Box 1, the classification of HCPs in the Netherlands is described according to the Netherlands Level Qualification Framework,^51^ which in turn is based on the European Qualification Framework.^52^ On all these levels, HCPs deliver palliative care.

**Box 1.** Classification levels of HCPs.

NLQF level	Healthcare professional
NLQF level 2 and 3	Nurse assistant
NLQF level 3+	Certified nurse assistant
NLQF level 4	Vocational level nurse
NLQF level 5 (before 2020) and 6 (after 2020)	Bachelor nurse
NLQF level 7	Clinical nurse specialist and physician assistant
NLQF level 8	Physician
NLQF level 8+	Specialized physician

HCP, healthcare professional; NLQF, Netherlands Level Qualification Framework.

The following interprofessional activities have been defined as EPAs.^36^ Each activity consists of a behavioral expression and a competency. They are based on the patient’s journey and relate to the patient’s disorder, symptoms, resilience, and context.

The EPA’s interprofessional collaboration model consists of the following activities:

1. Identifying, diagnosing, and indicating the palliative phase
2. Advance care planning
3. Performing and evaluating palliative care
4. Coordinating and continuity of palliative care
5. Indicating and guiding the dying phase
6. Providing aftercare.

Figure 3 shows the interprofessional collaboration model.

### Optimizing palliative care education (project 2)

Discussing what good palliative care education consists of created more awareness among teachers and educational developers within the education institutions. Furthermore, the first steps were taken to improve the presence of palliative care education within the curricula of participating institutions. This was done, for instance, by changing lessons, case studies, assignments, and assessments, and paying more attention to palliative care during lectures. The competencies described in the Education Framework for palliative care led to these improvements.

For instance, lessons about chronic illnesses, such as Chronic obstructive pulmonary disease (COPD) and heart failure, are no longer only about prevention and curation but now also include palliative care. In these lessons, prospective HCPs learn about the importance of talking to care recipients and loved ones about their future wishes and needs at an early stage, and about shared decision-making at the end of life. More attention is paid to future HCPs during return moments at school, especially their experience and reflection on caring for vulnerable care recipients.^11,53^

### Online education platform for palliative care (project 3)

The education platform is for all (future) HCPs searching for an overview of continuing education opportunities in palliative care. Other end users are trainers and teachers, who will find educational materials to integrate into their education or training programs on the platform. The following products on the platform consist of the measures described above. In addition, on the platform, there is an (A) toolbox with educational materials, (B) elective courses on palliative care for vocational training, (C) an overview of continuing education and training, and (D) a competency scan.

#### Toolbox

A toolbox on the education platform^54^ combines various educational materials and tools for further developing and improving specific palliative care education and training elements. It includes teachers’ manuals, PowerPoint presentations, case studies, and assignments and assessments for graduate students. The materials are freely available and linked to the Education Framework for palliative care competencies so they can be applied and implemented at the right level of education. A screening committee, consisting of members with expertise in palliative care education on all levels, assessed and approved the quality of the materials in the toolbox.

#### Elective course in palliative care for vocational training

Vocational level nurses choose an in-depth module in the final year of their training. The working group has developed a module in which elements of specialized palliative care are offered as a surplus of the basic competencies in initial education. This in-depth module can be implemented by vocational-level institutes in their educational programs. Competencies in leadership, providing complex palliative care, and using measuring instruments and reasoning aids are elements included in the elective course.^55^

#### Overview of continuing education and training

An extensive overview was made of regional and nationwide continuing education and training in palliative care.^56^ Users can search by filtering by subject, educational level, care setting, and regional location. New training courses are frequently added to this overview after screening by the screening committee. Criteria for inclusion in this overview are quality, relevance, applicability, and user-friendliness.

#### Competency scan

A competency scan was developed and made available on the platform for all HCPs. It consists of an online questionnaire linked to the CanMEDS roles,^57^ asking questions about knowledge, skills, and attitude.^58^ The scan identifies gaps in palliative care competencies and is linked to an overview of continuing education and training. It provides the user with personalized advice on education and training opportunities that could be suitable to overcome these gaps.

### Palliative care education hubs (Project 4)

The question remained how to ensure that students and HCPs know of and can enroll in local and regional palliative care education and training initiatives and that these initiatives align with national developments and innovations. To this end, a network of palliative care education hubs was developed.^59^

**Figure 4.**
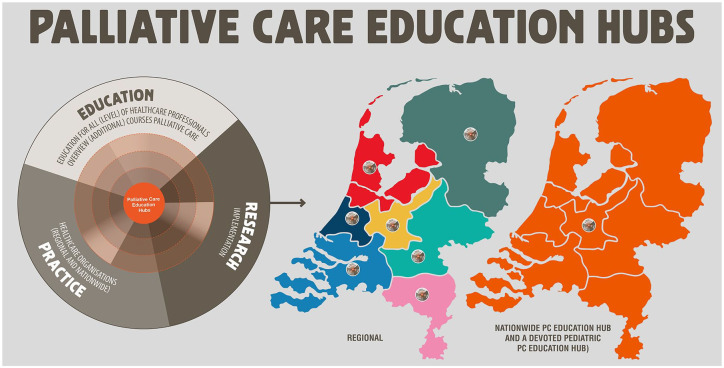
Palliative care education hubs.

In seven regions of the Netherlands, covering the entire country, a so-called education ambassador represents and leads the hub. These ambassadors are experienced educators in palliative care or experienced HCPs in palliative care with education affinity. Everything within a palliative care education hub is aimed at concretely implementing innovations and outcomes of research, developments, and activities in regional curricula. Hence, the network of hubs aims to bridge the gap between local and national activities and standards to prevent fragmentation and inequalities between regions. Furthermore, the hubs also focus on connecting education to regional research and healthcare practice. One strategy to connect this triad is to organize a webinar twice a year with regional speakers from education, research, and practice on a particular issue in palliative care. All educators and HCPs in the region are invited to participate. Figure 4 shows how the palliative care education hubs are organized in the Netherlands. In addition to the regional hubs, a national palliative care education hub connects all regional hubs, addressing common themes, policy developments, and innovations in education and palliative care practice. Another hub was established that is devoted to pediatric palliative care and covers all regions of the Netherlands.

## Discussion

Here, we discuss some challenges that we encountered in our projects. We will shortly address how these challenges were dealt with and discuss some lessons learned. This may be helpful to others who seek to improve palliative care education in their country.

### Building on what is already there

The O^2^PZ program was not the only initiative to improve palliative care education in the Netherlands; others also took initiatives, but mainly at a local or regional level,^9,29^ which caused fragmentation and inequalities in education opportunities among regions. However, we did not want to ignore or compete with these other initiatives. First of all, many of these initiatives were worthwhile in themselves. Second, ignoring or overruling them could have impeded building stakeholder trust and commitment, which is needed to implement and consolidate national education structures. Our challenge therefore was to take these individual initiatives into account, yet to go beyond merely collecting them and making them available at a national level.

To deal with this challenge, we used an *appreciative inquiry approach*.^35,60,61^ Concretely, this meant that we built new education structures while trying to integrate and improve what was already there in a step-by-step way. For instance, the education platform for palliative care is, for a large part, devoted to coherently presenting already existing educational tools and materials that have been shown to be successful and that came to meet the criteria of the evaluation committee that we installed.

### Dealing with blind spots and misconceptions

A major risk in developing the Education Framework for palliative care was that specific competencies (knowledge, skills, attitudes, and behaviors) were overlooked or misconstrued or that a professional level was misrepresented due to biases or a knowledge hiatus among the relatively small group of researchers and experts involved in establishing the framework. This could have compromised the framework’s applicability and feasibility in the designated education contexts, and end users may not have accepted it. However, by engaging a large group of researchers, an interdisciplinary group of HCPs, and other experts from the field to check the concept framework on omissions or misrepresentations, we were able to overcome bias or lacunas in the framework. Hence, developing the Education Framework for palliative care in a thoroughly participatory way was necessary to make it truly applicable and relevant to the various professional and educational levels of nursing and medical professionals. It also promoted wide support for the framework.

### Creating support, awareness, and commitment

Another challenge was establishing awareness of and commitment to the O^2^PZ program and its objectives among all relevant stakeholders. These stakeholders were most of all educators, policy-makers, students, and HCPs. Without such awareness and commitment, the results and products of the O^2^PZ program would not have any value for practice. We sought to engage people in various ways. First is by installing a steering board of stakeholders (see Figure 1), thus creating co-ownership among the “end users” of our products from the very start of the program.

In addition, we identified “early adaptors,” who embraced the program’s goals and accomplishments early on.^62,63^ We engaged them as contact persons within their educational institutions. Importantly, we sought to engage people involved in palliative care education at all levels: the policy level (funders, government, education councils), the organizational level (board and management of education institutions and healthcare organizations), and the operational or practical level (educators, students, HCPs, and coordinators of education programs).

Furthermore, we appointed a communication expert to share our achievements and developments with the outside world through a dedicated website, newsletters, and interviews, using social media such as X (formerly known as Twitter) and a LinkedIn page.

Finally, we organized an annual national symposium devoted to optimizing palliative care education to connect people from practice and education and facilitate exchange and mutual learning.

### Continuation of the O^2^PZ program

A major challenge that is still ongoing pertains to consolidating and continuing our work. This is the main objective of the present O^2^PZ program 2022–2025 projects, which follow up on the 2018–2021 projects.

The first project of the present follow-up program focuses on palliative care education for nurses at a master’s level, such as the master of advanced nurse practice or physician assistant. In the first project, we did not yet succeed in optimizing education palliative care in these curricula. Second, whereas the first program focused on palliative care education for generalists, a present study describes which competencies should be trained in curricula for palliative care specialists. Finally, a follow-up project focuses on consolidating the regional and national infrastructure of the educational hubs and on strengthening the position of the education hub ambassadors.

All in all, we have learned that sustainably improving palliative care education nationwide is more of a marathon than a sprint and more of a joint learning process than an individual accomplishment. It is, therefore, conducive to having sufficient funding and a dedicated team that can devote time and effort to facilitating this process for a more extended period.

## Conclusion

The O^2^PZ program 2018–2021 was dedicated to improving palliative care education nationwide. This task included the development of a national Education Framework for palliative care, including an interprofessional collaboration model, creating an online education platform, and establishing a network of palliative care education hubs. Ongoing follow-up studies will consolidate and elaborate on the initiatives taken. By discussing the O^2^PZ program, its methods and output, as well as some of its challenges and lessons learned, we sought to provide a practical example of how to improve palliative care education on a national level.

## Appendix

### List of abbreviations

EQF European Qualification Framework

HCPs Healthcare professionals

MANP Master of advanced nurse practice

NA Nurse assistant

NLQF Netherlands Qualification Framework

NQFPC Netherlands Quality Framework for Palliative Care

O^2^PZ Optimizing Education and Training in Palliative Care

PA Physician assistant

PC Palliative care

V&VN Dutch Nurses Association

WHO World Health Organization

## Declarations

**Ethics approval and consent to participate:** Not applicable. We performed our study by the relevant guidelines and regulations of the Declaration of Helsinki. According to the Medical Research Involving Human Subjects Act, committee ethics committee exempts our study from formal review (Central Committee on Research Involving Human Subjects, https://english.ccmo.nl). Participants were not asked to act or change behaviors; the questions were not drastic. According to Dutch law, no permission is required from an Ethic Committee, only a self-check of the Saxion Ethical Advisory Committee (SEAC).

**Consent for publication:** Not applicable.

**Availability of data and materials:** The data are available from the corresponding author on reasonable request.
